# Alterations of Dopamine D2 Receptors and Related Receptor-Interacting Proteins in Schizophrenia: The Pivotal Position of Dopamine Supersensitivity Psychosis in Treatment-Resistant Schizophrenia

**DOI:** 10.3390/ijms161226228

**Published:** 2015-12-17

**Authors:** Yasunori Oda, Nobuhisa Kanahara, Masaomi Iyo

**Affiliations:** 1 Department of Psychiatry, Chiba University Graduate School of Medicine, 1-8-1 Inohana, Chuou-ku, Chiba 260-8670, Japan; odayasunori@chiba-u.jp (Y.O.); iyom@faculty.chiba-u.jp (M.I.); 2 Division of Medical Treatment and Rehabilitation, Chiba University Center for Forensic Mental Health, 1-8-1 Inohana, Chuou-ku, Chiba 260-8670, Japan

**Keywords:** antipsychotic, dopamine signal, rebound psychosis, tardive dyskinesia

## Abstract

Although the dopamine D2 receptor (DRD2) has been a main target of antipsychotic pharmacotherapy for the treatment of schizophrenia, the standard treatment does not offer sufficient relief of symptoms to 20%–30% of patients suffering from this disorder. Moreover, over 80% of patients experience relapsed psychotic episodes within five years following treatment initiation. These data strongly suggest that the continuous blockade of DRD2 by antipsychotic(s) could eventually fail to control the psychosis in some point during long-term treatment, even if such treatment has successfully provided symptomatic improvement for the first-episode psychosis, or stability for the subsequent chronic stage. Dopamine supersensitivity psychosis (DSP) is historically known as a by-product of antipsychotic treatment in the manner of tardive dyskinesia or transient rebound psychosis. Numerous data in psychopharmacological studies suggest that the up-regulation of DRD2, caused by antipsychotic(s), is likely the mechanism underlying the development of the dopamine supersensitivity state. However, regardless of evolving notions of dopamine signaling, particularly dopamine release, signal transduction, and receptor recycling, most of this research has been conducted and discussed from the standpoint of disease etiology or action mechanism of the antipsychotic, not of DSP. Hence, the mechanism of the DRD2 up-regulation or mechanism evoking clinical DSP, both of which are caused by pharmacotherapy, remains unknown. Once patients experience a DSP episode, they become increasingly difficult to treat. Light was recently shed on a new aspect of DSP as a treatment-resistant factor. Clarification of the detailed mechanism of DSP is therefore crucial, and a preventive treatment strategy for DSP or treatment-resistant schizophrenia is urgently needed.

## 1. Introduction

Schizophrenia is a serious mental disorder that generally occurs in young people from late adolescence to early adulthood and profoundly burdens patients throughout their lives. Since the 1950s, antipsychotics have been a mainstay in treating this disorder. The beneficial effects of these agents, however, are generally restricted. This is true even though the type of agents with the common action mechanism of the blockade of the dopamine D2 receptor (DRD2), which is the action mechanism common to this type of agents, ameliorates symptoms in most patients. Since functional recovery has been recognized as a main treatment goal for all patients suffering this disorder, greater attention has been paid to relief of negative symptoms or cognitive impairments. Thus, recently, the development of novel agent targeting receptors other than DRD2 has been viewed as crucial. However, it is still necessary to block DRD2 when treating this disorder, since both first-episode and relapsed psychosis generally appears with positive symptoms in most patients, and physicians must address such situations with antipsychotics. On the other hand, it has been reported that the blockade of DRD2 is directly involved in several distressful adverse events, including extrapyramidal symptom, hyperprolactinemia, cognitive dysfunction, and dysphoria. “Appropriate” blocking level of DRD2 on the part of every antipsychotic is therefore imperative to physicians. Maintaining such a blockade, however, is a very difficult task, given that the natural clinical course is quite complex, varying from patient to patient.

Dopamine supersensitivity psychosis (DSP) is both an old and new concept in schizophrenia. Although tardive dyskinesia (TD), a classical neurological sign of DSP, is generally recognized to be a result of continuous pharmacotherapy, DSP related to other symptomatology, such as a withdrawal psychotic episode or developed tolerance to the effects of antipsychotic(s), do not simply indicate a side effect in pharmacology. However, the appearance of DSP has a definite negative impact on long-term prognosis. Thus, clarifying the mechanisms of DSP development and establishing preventive strategies for it have become an increasingly important subject.

This review will touch first on the clinical concept of treatment-resistant schizophrenia (TRS) and DSP. Next, we will focus on possible mechanisms of DSP, particularly from the perspective of the up-regulation and presumed accompanying impairment of cycling of DRD2 caused by antipsychotics. To discuss these issues, we will summarize various datasets of DRD2 itself, as well as several receptor-interacting proteins such as G protein-coupled receptor kinase (GRK), β-arrestin, dopamine-and cyclic adenosine monophosphate (cAMP)-regulated phosphoprotein of 32 kDa (DARPP-32), protein kinase v-akt murine thymoma viral oncogene homolog AKT, glycogen synthase kinase-3 (GSK-3), and clathrin, tracing these from the basic and clinical research fields relevant to schizophrenia and the actions of antipsychotics. This will allow us to discuss a possible mechanism for the development of refractory schizophrenia.

## 2. Clinical Concept of Dopamine Supersensitivity Psychosis

### 2.1. Dopamine Supersensitivity Psychosis in the Course of Schizophrenia

Antipsychotics act mainly on the DRD2 in brains of patients with schizophrenia, and provide symptomatic relief, particularly for positive symptoms [1]. However, while 20%–30% of patients can attain remission [2,3], from one-fifth to one-half of patients eventually develop a treatment-resistant state [4].

The clinical course of schizophrenia generally varies from patient to patient. Numerous parameters predictive of prognosis have been examined from multiple viewpoints: male gender [5], younger disease onset [6], presence of family history of psychiatric disease [7], longer duration of untreated psychosis [8,9], and lower premorbid social function [10]. There is, however, no single factor that completely predicts the prognosis of patients with different etiologies. It seems that several clinical factors could affect the disease’s severity or course and contribute to the development of treatment-resistance, with interaction between individual factors.

Several studies have reported that 12%–15% of patients show little response to initial pharmacotherapy for their first psychotic episodes [11,12,13]. This is clearly a lower percentage of “non-responders” than that of treatment-resistant patients after long-term treatment. These ratios imply that some portion of patients respond well to pharmacotherapy in the early stage but become poor responders to subsequent treatment. It is therefore suggested that responsiveness to antipsychotics could alter over the clinical course or with continuous treatment.

Cases where psychotic symptoms worsened immediately upon cessation of treatment and thereafter required gradually higher dosage of drugs to control psychosis were reported in the late 1970s [14,15,16]. It was suggested that the clinical phenomena of increasing severity of psychosis was supersensitivity psychosis, which could latently develop during pharmacotherapy and be covertly masked with antipsychotics but overtly appear upon dose reduction or discontinuation of treatment [17]. This concept, recently relabeled DSP [18], includes several clinical elements: (1) withdrawal or rebound psychosis: relapsed psychotic episode appears immediately after treatment cessation, reduced dosage or change of antipsychotic [19]; (2) developed tolerance to antipsychotic effect: higher dosage of antipsychotic(s) are needed to control the psychosis, or perhaps not even high-dosage treatment can control the psychosis [20]; (3) TD: a classical neurological sign of dopamine (DA) supersensitivity, which occurs in the nigrostriatum pathway of dopaminergic neurons; and (4) increasing vulnerability to stress [21]; (5) DA partial agonist-induced exacerbation: administration of DRD2 partial agonist, aripiprazole, exacerbates or relapses psychosis [22]. It has been established that the blockade of DRD2 in post-synaptic neurons by antipsychotics plays an essential role in controlling psychosis, and presumed that the supersensitivity of the receptors caused by the antipsychotic blockade concurrently underlies the development of DSP. Therefore, DSP can appear in a number of clinical manners, not in a single symptom domain, and can manifest symptoms similar to the original psychotic symptoms.

Few data exist on the occurrence ratio of any specific antipsychotic. Several reports and studies suggested that agents with a short half-life or lower binding affinity to DRD2 were potentially related to the occurrence of DSP [23,24]; this might be interpretable as such agent types succumbing to endogenous DA. Iyo *et al.* argued that agents with a longer half-life should be applied to treat patients who have already developed DSP, and actually demonstrated that this type of agent (*i.e.*, a long-acting injectable form) was effective for such patients [25].

### 2.2. Dopamine Supersensitivity Psychosis in Treatment-Resistant Schizophrenia

DSP, particularly withdrawal psychosis, has sometimes been viewed only as a transient relapse, like “hump psychosis” [26], related to dose reduction or switching of antipsychotic. However, it is increasingly being shown that DSP could be linked to etiology in treatment-resistant schizophrenia. Several earlier retrospective studies reported that only a few patients (4.5%–22%) experienced such an episode in the course of their treatment [27,28]. It could be more difficult for patients with TD to recover from psychotic relapse than for those without TD, indicating a readiness for treatment-resistance [29]. Furthermore, there was little data on developed tolerance to the effects of antipsychotics.

Chouinard and Chouinard estimated that half of TRS cases could be etiologically related to DSP, based on the relatively high ratio of TD reported in numerous clinical trials with conventional antipsychotics [30]. We recently evaluated the frequency ratio of any DSP episode for 147 TRS patients through interviews and clinical records [31]. The results revealed that 72% of the patients had experienced an episode of withdrawal psychosis, developed a tolerance to an antipsychotic, or had TD over the course of their treatment histories of over 20 years. The large differences in the frequency ratio of DSP episodes in other studies might be attributable to their methodologies, particularly in selecting relevant episodes. It also suggests that the appearance of DSP could vary depending on patients’ severity and treatment state. In addition, our survey showed that the ratio of withdrawal psychosis, developed tolerance to antipsychotic and TD was 41.5%, 55.7% and 44.3%, respectively, indicating that DSP types were distributed with similar ratios. These results suggest that DSP could manifest diverse symptoms to varying degrees.

Although these notions suggest that DSP plays some contributing role in the development of TRS, it seems to be difficult to measure the degree of its effect with any objective parameter, such as effect size. This is because many other clinical factors, including gender, family history of psychiatric disease, age at onset, duration of untreated psychosis, and substance abuse, as well as the interaction of these factors, have also been traditionally raised as contributors to TRS. Additionally, it is sometimes impossible to distinguish a rebound psychosis as DSP rather than as general true relapse episodes, which could occur in up to 50% or more of patients mainly due to noncompliance or rejection of treatment [32]. As a matter of fact, data of relapse rates in schizophrenia might include the significant amount of rebound psychosis, which it would be strictly appropriate to judge as DSP [33].

Contrary to the number of case reports and clinical surveys on DSP, control-designed studies on specific types of agent are rare [19]. In addition, there has never been a study directly measuring *in vivo* DRD2 within a patient’s brain during a DSP episode. DSP, therefore, has not yet been demonstrated clinically in humans. Furthermore, it is unknown whether rebound psychosis of DSP and true relapse share any common pathway to the development of TRS at the biological level. Hence to clarify the etiology of recurrent psychosis, it might be important to explore these two phenomena separately as strictly as possible.

## 3. Biological Background of Dopamine Supersensitivity Psychosis

### 3.1. Dopamine D2 Receptor

#### 3.1.1. Up-Regulation of Dopamine D2 Receptor in Animal Models and Patients with Schizophrenia

DRD2 is distributed at high levels in the striatum, nucleus accumbens and olfactory tubercle, as well as in the dopaminergic neurons in the mesolimbic pathway projecting from the ventral tegmental area to nucleus accumbens, which is closely related to positive symptoms in schizophrenia. All antipsychotics are recognized to have ameliorating effects on positive symptoms via their blockades of post-synaptic DRD2 [1].

A number of basic studies have demonstrated that a blockade of DRD2 by antipsychotics, typical and atypical, leads to a compensatory increase in DRD2 densities [34,35,36,37,38,39], and this phenomenon is presumed to be, at least partly, related to the development of the DA supersensitivity clinically observed as DSP in patients with schizophrenia. Interestingly, there are some reports that DRD2 densities in rats barely increase during administration of antipsychotics, but gradually increase over a week after cessation [37,38]. According to the clinical findings, these results might elucidate the mechanism of rebound psychosis. On the other hand, Seeman and colleagues demonstrated that although amphetamine-sensitized rats underwent very little change in absolute DRD2 density, they exhibited greater dopaminergic hyperlocomotion on the behavioral level. It is possible for DRD2 to have a high affinity (D2^High^) or low affinity (D2^Low^) for DA. Seeman *et al.* explained the observed phenomenon as an almost 4-fold D2^High^ increase in amphetamine-sensitized rats compared to controls [40]. Additionally, Seeman *et al.* reported that subchronic antipsychotic administration of an appropriate dose occupied up to 70% of the striatum DRD2 and induced an approximate doubling of D2^High^ [41]. Therefore, Seeman considered that the mechanism of DSP is essentially associated not with total DRD2 but with D2^High^.

Similarly, postmortem studies of patients’ brains revealed that the DRD2 density was elevated only about 1.4-fold in the striatum of patients with schizophrenia, compared to controls with no psychiatric disorders [42], although such studies generally included patients experiencing a variety of antipsychotic treatments. To date, there has never been a study focusing on the direct effects of a specific class of the agent on DRD2 density. Thus, it is impossible to evaluate the state of supersensitivity of DRD2, or the degree of DSP, from data only of the density of DRD2 in postmortem brain studies.

The Positron Emission Tomography (PET) and Single Photon Emission Computed Tomography (SPECT) research fields of DRD2 measurement in *in vivo* human brains are also inherent to overcoming the difficulties in interpreting the direct effect of antipsychotics on receptor density with results showing the binding potential of radioligands. While several studies have shown that DRD2 density in patients under chronic antipsychotic treatment tended to be higher than that of drug-naïve patients [43], this was not the case in all studies [44,45]. When these similar studies are considered together, it is estimated that the increase rate of DRD2 availability in the striatum is relatively small (mean of 5.8%–12%) [46,47] or moderate [48,49]. These human neuroimaging data seem to be relatively consistent with the overall data in other research fields like animal models and postmortem studies.

There have been several PET studies focusing on measurement of the D2^High^ state in schizophrenia. The ^11^C-(+)-4-propyl-9-hydroxynaphthoxazine (^11^C-(+)-PHNO) tracer used in these studies binds both the DRD2 and DRD3, with a higher affinity for D2^High^ or DRD3. Graff-Guerrero *et al.* [50] reported that their finding was no increase in D2^High^ or DRD3 in any brain region of the patient group compared to the healthy control group. However, the researchers suggested an inaction mechanism of antipsychotics on DRD3 in the globus pallidus might be important [51]. Several PET studies on the modulation of DA depletion by α-methyl-*para*-tyrosine (α-MPT) or amphetamine showed higher DRD2 availability at baseline in patients compared to controls, suggesting increased DA signal transduction [52,53,54]. Additionally, TRS patients were shown to have lower capacity of DA synthesis by the measured uptake of ^18^F-dihydroxyphenylalanine (DOPA) compared to both treatment-responsive patients and healthy controls [55]. This suggests some disruptions in presynaptic dopaminergic neurons as the essential pathophysiology of refractory symptoms. PET studies with more sophisticated techniques, or for more clinically homogeneous samples, could provide more detailed insight into presynaptic alterations, including the synthesis and release of DA, in addition to or instead of postsynaptic DRD2 alterations. However, given that previous studies on the frequency of DSP among patients indicated that these episodes were not observed in all patients, it is possible that such PET/SPECT studies provide results biased toward stable subjects, excluding patients with DSP.

#### 3.1.2. Effects of Genetic Polymorphisms of Dopamine D2 Receptor

Numerous studies have suggested that several genetic polymorphisms of DRD2 are related to the receptor density in human brains. Taq I A (rs1800497: C>T) is one of the most extensive targets in this research field. Taq I A exists in the *ankyrin repeat and kinase domain containing 1* (*ANKK1*) gene, which is located approximately 10 kb downstream of the *DRD2* gene [56]. The frequency of a minor allele having a T substitution, called the A1 allele, is 20%–44%, depending on ethnicity, whereas the major allele having a C allele is called the A2 allele. In healthy human subjects with A1 allele, the DRD2 density was reported to be lower than that of subjects without this allele in several PET studies [57,58]; however, postmortem studies showed the reverse [59,60]. −141 C·Ins/Del (rs1799732), located at the 5′-promotor region of *DRD2*, is another important functional genetic polymorphism [61]. The minor allele (deleting C substitution) significantly differs in frequency among ethnicities, with a range of 10%–50%. Whereas the Del carrier shows a lower expressed level of the receptor in cultured cells [61], an *in vivo* PET study demonstrated contrasting results; the Ins/Ins carrier showed lower DRD2 density [58].

Furthermore, it was reported that these polymorphisms are related to responsiveness to antipsychotic treatment in patients with schizophrenia. As regards Taq I A, homozygous A2/A2 (A1(−)) carriers showed poorer response compared to A1/A2 carriers [62]. Regarding −141 C, Del carriers responded poorly to treatment [63,64]. A meta-analysis demonstrated that Del carriers had poorer responses than subjects with the Ins/Ins homozygous genotype, whereas Taq I A genotypes did not differ in antipsychotic responsiveness between A1/A1 and A2 carriers [65]. When analyzed together with both polymorphisms, subjects with A1(−) +Del alleles could be the poorest genotypes [66,67]. Taken together with previous intensive studies on several SNPs (single nucleotide polymorphisms) on *DRD2* gene, the results of the Del allele of −141 C would be highly consistent in terms of the poor response to antipsychotic. Several cultured cell and *in vivo* PET studies have suggested that the SNP could affect the expression level of DRD2, although not necessarily in the same direction. Therefore, it remains to be clarified as to whether the aberrant expressed level of DRD2 is actually observed within brains of patients who are Del carriers, particularly under the administration of antipsychotics. The question of whether the Del allele, potentially related to poor response, is further related to DSP remains unresolved.

### 3.2. Downstream of the Dopamine D2 Receptor

#### 3.2.1. Dopamine D2 Receptor Signaling

**Figure 1:**
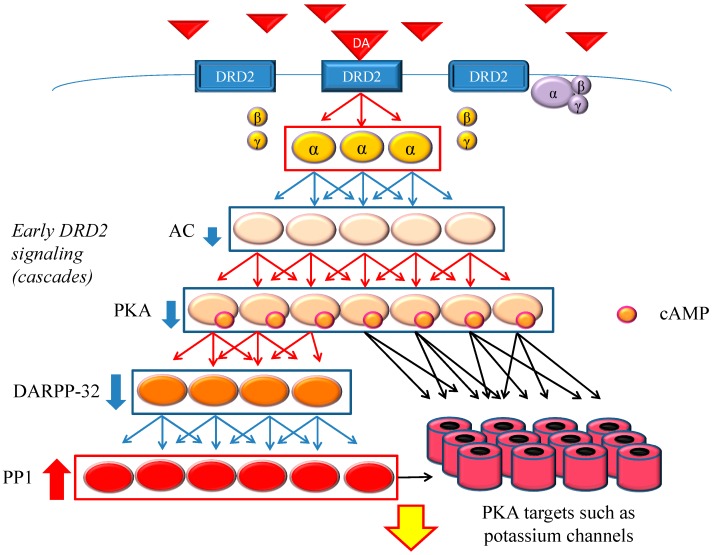
Dopamine D2 receptor (DRD2) early signaling. The stimulation of DRD2 by agonists induces early and late signals. In the early phase, acute and evanescent G protein-mediated signaling occurs. This signaling is amplified at several stages of the cascade. As a result, the function of phosphorylation of protein kinase A (PKA) and DARPP-32 is suppressed, and PP1 is activated. The heavy up-pointing red arrows indicate functional facilitation, and the heavy down-pointing blue arrows indicate functional suppression. The thin red arrows indicate activation and the thin blue arrows indicate inactivation. The black arrows represent either activation or inhibition of each specific substrate. The yellow arrow with red frame border indicates DRD2 early signaling. The α, β and γ indicate each subunit of G proteins. The yellow α represents guanosine triphosphate (GTP) type, while the purple one represents guanosine diphosphate (GDP) type. Abbreviations: AC, adenylate cyclase; DA, dopamine; DARPP-32, dopamine- and cyclic adenosine monophosphate (cAMP)-regulated phosphoprotein of 32 kDa; DRD2, dopamine D2 receptor; PKA, protein kinase A; PP1, protein phosphatase 1.

**Figure 2:**
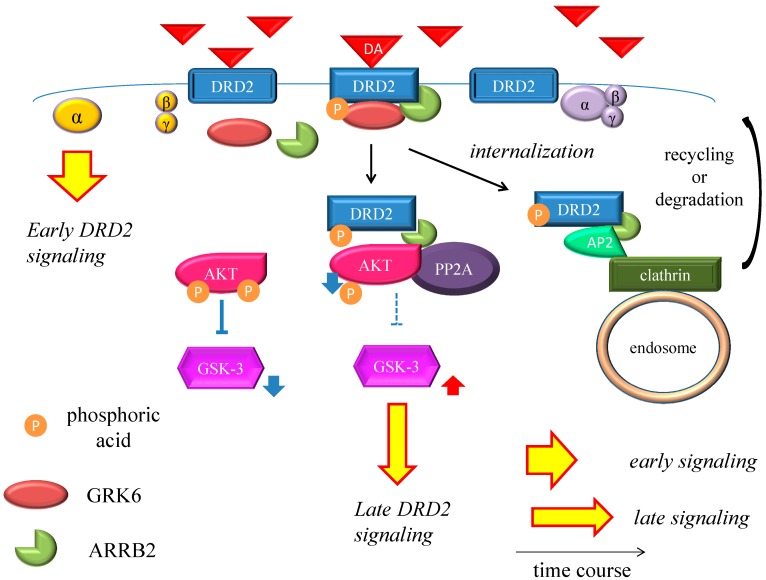
Dopamine D2 receptor late signaling. In the late phase, GRK6 phosphorylates the stimulated DRD2, and then ARRB2 binds to the phosphorylated receptors and prevents further G protein activation. Subsequently, the DRD2 binding to the ARRB2 promote the formation of the ARRB2/PP2A/AKT complex, resulting in the deactivation of AKT and stimulation of GSK-3 signaling. This G protein-independent signal is more moderate and longer lasting than the early signal. On the other hand, DRD2 is internalized from the cell membrane and experiences CME. Finally, the internalized receptors recycle back to the cell surface or degrade. The straight black arrows indicate the process to form the AKT/PP2A/ARRB2 complex or internalization of DRD2. The arcate black arrow indicates recycling or degeneration of DRD2. The up-pointing red arrows indicate functional facilitation, and the down-pointing blue arrows indicate functional suppression. The blue T-arrows indicate inhibition and the dashed T-arrows indicate disinhibition. The short yellow arrow with red frame border and long one indicate early signal and late signal, respectively. Abbreviations: AKT, protein kinase v-akt murine thymoma viral oncogene homolog; AP2, adaptor protein; ARRB2, β-arrestin 2; CME, clathrin-mediated endocytosis; DA, dopamine; DRD2, Dopamine D2 receptor; GSK-3, glycogen synthase kinase-3; GRK6, G protein-coupled receptor kinase-6.

DRD2 belongs to the G protein-coupled receptors (GPCRs) superfamily, which consists of the most diverse subgroups among proteins involved in transmembrane signaling. G proteins are generally composed of three types of subunits (α, β, and γ). After the agonists bind to DRD2, two different signals occur, *i.e.*, early and late signals [68] (Figure 1 and Figure 2). In the early phase, the binding of the agonist to DRD2 induces the activation of G proteins. The α subunit then changes its guanosine diphosphate (GDP) to guanosine triphosphate (GTP), and is dissociated from the βγ-complex. The α subunit with GTP inhibits activity of adenylate cyclase (AC) and provokes an acute and evanescent change in the phosphorylation of protein kinase A (PKA), which targets dopamine-and cAMP-regulated phosphoprotein of 32 kDa (DARPP-32) and others. On the other hand, in the late phase, following the activation of DRD2 by the agonist, G protein-coupled receptor kinase 6 (GRK6) phosphorylates the receptors, and then β-arrestin 2 (ARRB2) binds to the phosphorylated receptors and prevents further G protein activation. After this process, the DRD2 binding to ARRB2 promotes the formation of ARRB2/protein phosphatase 2 (PP2A)/protein kinase v-akt murine thymoma viral oncogene homolog (AKT) complex, resulting in the deactivation of AKT and subsequent stimulation of glycogen synthase kinase-3 (GSK-3) signaling [69]. This signal is more moderate and longer lasting than the early signal. In addition, DRD2 is internalized from the cell membrane, and experiences clathrin-mediated endocytosis (CME). Finally, the internalized receptors recycle back to the cell surface or degrade [70,71,72,73].

#### 3.2.2. Dopamine- and Cyclic Adenosine Monophosphate (cAMP)-Regulated Phosphoprotein of 32 kDa (DARPP-32)

DARPP-32 acts as an inhibitor of protein phosphatase-1 (PP-1) in the early phase of DA signaling [74]. It also serves as a molecular integrator of dopaminergic and glutaminergic signaling [75]. Following phosphorylation at threonine (Thr) 34 of DARPP-32 by PKA, the phosphorylated DARPP-32 inhibits PP-1 [74], while dopamine D2-class signaling dephosphorylates DARPP-32 through inhibition of PKA and activation of calcium/calmodulin-dependent protein phosphatase [76]. It is widely expressed throughout many brain areas, particularly in the caudate nucleus, putamen, nucleus accumbens, and cerebellar cortex of the primate [77,78].

With regard to animal studies, DARPP-32 knockout (KO) mice did not show d-amphetamine-disrupted prepulse inhibition [79]. They also exhibited response deficits to moderate doses of cocaine [80]. Therefore, it is conceivable that these mice exhibited little response to DA. Previous basic studies on DARPP-32 strongly suggested that this molecule has an intimate involvement in DA signaling.

*DARPP-32* has two major splice variants, *i.e.*, the full-length protein isoform (FL-DARPP-32) and a truncated isoform (*t*-DARPP-32) [81]. Interestingly, *t*-DARPP-32 lacks the NH_2_-terminal of the Thr34 phosphorylation site. PKA could not phosphorylate *t*-DARPP-32, and thus *t*-DARPP-32 could fail to work the dopaminergic signaling pathway normally. Kunii *et al.* reported that *t*-DARPP-32 expression was higher in the postmortem striatum of patients with schizophrenia [82]. On the other hand, it has been reported that there were no significant differences in four SNP distributions of *DARPP-32* between Japanese patients with schizophrenia and control groups [83].

Although previous studies have suggested that DARPP-32 is related to schizophrenia, whether its role is essentially involved in the disease etiology remains unclear. Since DARPP-32 plays a crucial role in the early dopamine signal, its role in the action mechanism of antipsychotics must be clarified in further studies, and its involvement in developing the DA supersensitivity state might be considered.

#### 3.2.3. G Protein-Coupled Receptor Kinase 6 (GRK6)

The function of GRKs is generally to phosphorylate the activated GPCRs and desensitize it. Seven isoforms have been identified in the GRK family [84]. Of these, GRK6 is widely expressed throughout the brain; its expression level is particularly high in the striatum, much higher than those in other regions [85,86]. GRK6 is located predominantly in dopaminergic neurons [86] and functionally phosphorylates DRD2.

Given GRK6 is functionally indispensable for DRD2 internalization by ARRB2, malfunction of GRK6 may induce DRD2 up-regulation or even DSP. In fact, GRK6-KO mice exhibit a significantly increased locomotor response to psychostimulants such as cocaine and amphetamine [86,87]. Furthermore, overexpression of GRK6 in rats and macaques promoted the internalization of DRD2 and improved l-DOPA-induced dyskinesia, possibly by preventing an excessive G-protein signal [88]. GRK6 could thus play a possible role in the development of DA supersensitivity.

There have been two studies of the genetic association of *GRK6* in patients with schizophrenia. The first reported no significant difference in any SNP distributions between case and control groups [89]. The second study, focused on DSP itself, showed no significant differences between cases and controls, or between patients with or without a history of DSP [90]. Some studies have reported that GRK6 expression level did not differ in the postmortem cortex in patients with schizophrenia, compared to control subjects without psychiatric disease [91,92]. To date, however, there have been no studies measuring GRK6 expression level for specific subjects with DSP.

In short, from animal studies it is considered that GRK6 is likely related to the formation of DA supersensitivity, but data for human or actual patients are quite scarce.

#### 3.2.4. β-Arrestin 2 (ARRB2)

ARRBs consist of ARRB1 and ARRB2. Although ARRB1 is a major subtype in the rat brain, ARRB2 has an approximately 6-fold greater affinity than ARRB1 for clathrin, which is involved in the internalization of receptors to endosomes [93]. ARRB2 not only internalizes DRD2 phosphorylated by GRK6 but activates G protein-independent cellular signaling mediated by the AKT/PP2A/ARRB2 complex, resulting in slower and longer-lasting signaling than G protein-mediated early signaling [72,94,95,96,97]. In addition, it has been reported that that the absence of ARRB2 not only promotes G protein-mediated early signaling, but actually disrupts the interaction of AKT with PP2A (*i.e.*, G protein-independent signaling). Moreover, ARRB2-biased partial DRD2 agonists have been shown to inhibit phencyclidine-induced hyperlocomotion in wild-type mice, and this effect is lost in ARRB2-KO mice [98]. These findings indicate that ARRB2 signaling may play an important role in the DRD2-mediated effects of antipsychotics [98].

There are only two studies about the genetic association between *ARRB2* and schizophrenia. The first reported no significant difference between patients with schizophrenia and controls [99], while the second examined the association between rs1045280 and TD induced by antipsychotics in patients with schizophrenia, whereby the T allele in rs1045280 was a risk allele for TD [100].

Seeman recently suggested that GRK6 and ARRB2 could regulate DRD2 in a state of low-affinity for DA (D2^low^) [101]. We also demonstrated that striatal ARRB2 in DSP model rats tended to decrease, and the ratio of GRK6/ARRB2 in DSP model rats was significantly higher than that in controls [102]. These results indicate that the GRK6/ARRB2 system was altered and could not perform DRD2 internalization normally in DSP rats. Given that ARRB2 is involved not only in DRD2 in internalization but also in the DRD2-mediated effects of antipsychotics, it is thus possible that the alteration of the GRK6/ARRB2 system has an impact on the pathogenesis of DSP. However, the relationship between the GRK6/ARRB2 system and actual patients with DSP is less well understood. Thus, further detailed research is necessary.

#### 3.2.5. Protein Kinase v-akt Murine Thymoma Viral Oncogene Homolog (AKT)

AKT serves many functions, such as protein synthesis, cell survival, cell proliferation, and glucose metabolism. It consists of three isoforms (AKT1, AKT2 and AKT3). Following dephosphorylation of AKT by DA stimulation, AKT activity decreases and GSK-3 is activated [69].

AKT1-KO mice exhibited enhanced psychotomimetic response to amphetamine and ketamine [103,104]. Additionally, female AKT-KO mice were insensitive to clozapine, and their PPI deficits were partially ameliorated by inhibitor of GSK-3 which lies downstream of AKT [105]. Interestingly, lithium inhibited the formation of ARRB2/PP2A/AKT, leading to augmented AKT activity. Lithium thus reduced G protein-independent cellular signaling by inhibition of GSK-3β [69]. Consequently, lithium may inhibit dopaminergic behavior by inhibiting GSK-3β. It follows that AKT regulates GSK-3-mediated G protein-independent signaling. Therefore, depressed AKT function may cause DA supersensitivity state.

A number of studies have reported a genetic association between AKT1 and schizophrenia [100,106,107,108,109]; other studies failed to support these results. A meta-analysis of AKT1 has demonstrated that rs2494732 (A>G) located at 3′-UTR of the *AKT1* gene was significantly associated with schizophrenia [110]. Importantly, rs2494732 has an effect on the progression of psychotic disorders caused by cannabis [111,112]. Moreover, it has been shown that the expressed protein levels of AKT1 significantly decreased in the postmortem frontal cortex and hippocampus of patients with schizophrenia compared to controls [103].

These findings imply that impaired AKT1 activity may be involved in development of schizophrenia, and could be a potential therapeutic target, since AKT1 regulates G protein-independent signaling of DRD2 [113].

#### 3.2.6. Glycogen Synthase Kinase-3 (GSK-3)

GSK-3 is multifunctional serine/threonine kinase, and plays important roles in cell proliferation, apoptosis, and development. GSK-3 consists of two isoforms, GSK-3α and GSK-3β. GSK-3 is highly abundant in the brain [114,115]. As described above, DRD2 stimulation by DA inhibits AKT, which suppresses GSK-3β activity and thus makes GSK-3β more active [68,69].

Dominant negative GSK-3 transgenic mice exhibited depression of DRD2-mediated function in the striatum [116]. In addition, GSK-3β inhibitors attenuated psychotomimetic effects induced by ketamine [117]. On the other hand, GSK-3β overexpression in mice showed an electroretinogram anomaly similar to the finding in subjects with high genetic risk for schizophrenia [118]. Moreover, prenatal polyriboinosinic-polyribocytidilic acid-induced neuropsychiatric disease model mice exhibited reduction of AKT and increase of GSK-3β in the medial prefrontal cortex [119]. In light of pharmacotherapy, a high dose of haloperidol or risperidone induced increase in GSK-3 of the rat striatum, and this finding was suggested as a therapeutic effect [120]. However, given that the excessive blockade of DRD2 induced DA supersensitivity in the rats, and that GSK-3β inhibitors attenuated the psychotomimetic effects induced by ketamine [117], the increase of GSK-3 could instead be a by-product of treatment with antipsychotics, conceivably implying one of the pathogenic mechanisms of DSP. Taken together, GSK-3 plays a central role in G protein-independent DRD2 signaling. Considering the compensatory increase of GSK-3 after administration of high dose antipsychotics, up-regulation of GSK-3 signaling may be responsible mechanism of DSP. In fact, convergent evidence indicates that the ARRB2/AKT/GSK-3 signaling pathway plays a crucial role in the regulation of DA mediated responses [68,121,122,123,124].

Some studies have demonstrated an association between *GSK-3β* polymorphisms and diagnosis of schizophrenia [125,126,127]. Furthermore, a systematic meta-analysis showed that rs334558, functional polymorphism in the *GSK-3β* promotor region, is associated with incidence of schizophrenia, and suggested that this SNP might be used for differential diagnosis between schizophrenia and bipolar disorder [128]. Kozlovsky *et al.* reported that both protein and mRNA of GSK-3β were decreased by approximately 40% in the postmortem frontal cortex of patients with schizophrenia relative to patients with bipolar or unipolar mood disorders and normal controls [129,130]. However, data in the postmortem striatum is lacking.

As presented above, it is estimated that GSK-3 has tremendous significance in the DSP mechanism. Therefore, it is strongly hoped that more studies of this molecule combined with detailed clinical parameters on symptomatology or pharmacotherapy will proceed.

#### 3.2.7. Clathrin

DRD2 internalization is regulated by CME [131] as other receptors such as α-amino-3-hydroxy-5-methyl-4-isoxazolepropionic acid receptor (AMPA), and γ-aminobutyric acid A (GABA(A)) are regulated by this mechanism [132,133]. Therefore, alteration of clathrin-interacting proteins (e.g., adaptor protein 2, Stonin 2, Epsin 4 and others) could also be involved in the pathophysiology of schizophrenia [73]. However, whether CME relates to the DA superesensitivity state or not is less well understood.

### 3.3. Other Pathways in the Process of Tardive Dyskinesia (TD) Development

Although it has been established that DRD2 supersensitivity acquired by use of antipsychotics was related to the occurrence of TD, other neuronal damage caused by antipsychotics has also been considered. Antipsychotics are transmitted through the membrane of nigral neurons and accumulate in a complex form with neuromelanin within the cells. This phenomenon could promote the denervation of dopaminergic neurons and the up-regulation of DRD2. It is possible that metoclopramide causes these processes more readily than other antipsychotics, which is consistent with the high frequency of TD in humans by metoclopramide administration [134,135,136].

### 3.4. Hypothesis of Dopamine Supersensitivity Psychosis (DSP) Mechanism

As we reviewed the relevant research on DRD2 and its interacting molecules for this paper, we found few studies that addressed the question of why antipsychotics could cause an up-regulation of DRD2 or whether the up-regulation of DRD2 actually underlies DSP. Nevertheless, we are particularly focused on these molecules, and now present a theory on the mechanisms of DSP.

**Figure 3:**
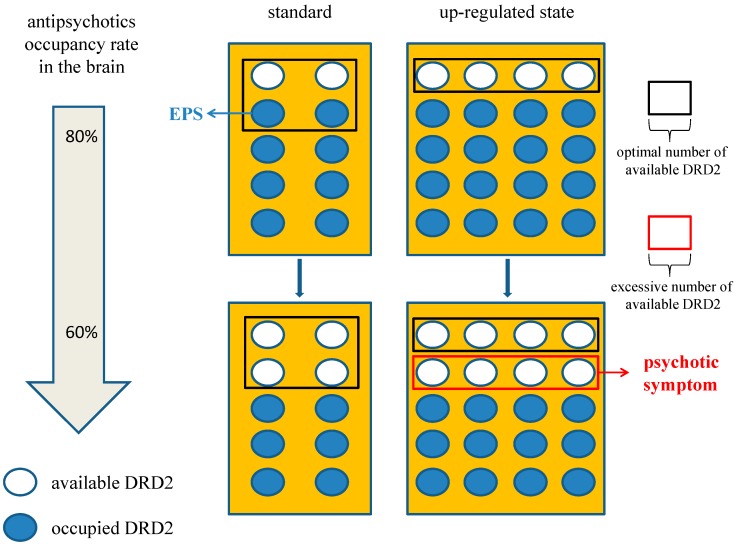
Effect of antipsychotic brain level on dopamine supersensitivity psychosis (DSP). If we set the condition that four DRD2 is the optimal number to maintain appropriate signaling, 80% occupancy of DRD2 in the up-regulated state is an adequate blockade. However, it could induce EPS in the standard state for excessive blockade. In the same way, 60% occupancy in up-regulated state could provoke psychosis relative to the adequate blockade in the standard state. Therefore, patients with DSP may be more affected by a reduction or elimination half-life of antipsychotics. The number of DRD2 in the area framed with the black box is optimal one and that with the red box is excessive available one which could induce psychotic symptom. The white circles indicate available DRD2 and the blue circles indicate DRD2 occupied by antipsychotics. Abbreviation, DRD2, dopamine D2 receptor; EPS, extrapyramidal symptom.

**Figure 4:**
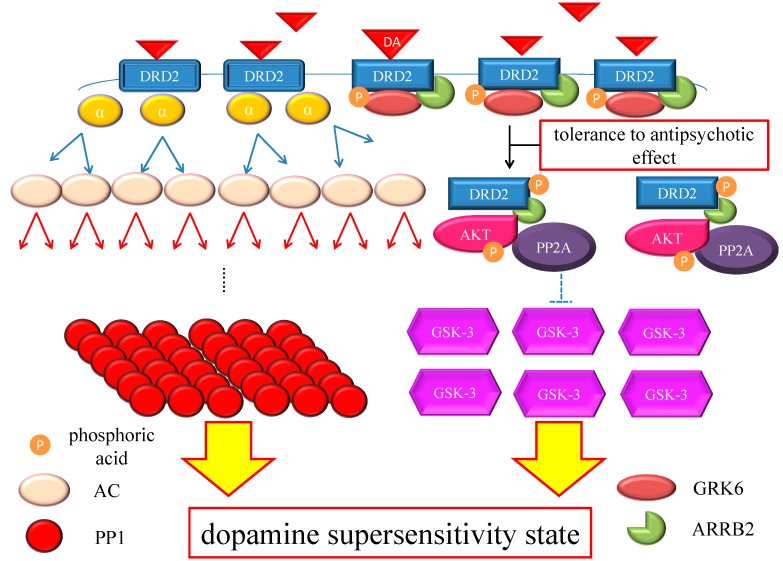
Hypothesis of DSP mechanism. Chronic excess blockade of DRD2 by antipsychotics induces vicarious increase in DRD2 densities. Following DRD2 up-regulation, G protein-mediated early signaling is enhanced. On the other hand, the alteration of the GRK6/ARRB2 system could induce tolerance to the antipsychotic effect. Moreover, GSK-3 also experiences compensatory up-regulation. Therefore, both early and late DRD2 signaling becomes strong, ultimately resulting in DSP. The red arrows indicate activation and the blue arrows indicate inactivation. The dashed T-arrows indicate disinhibition. The heavy yellow arrow with red frame border represents enhanced signal. The black arrow indicates the several processes to form the AKT/PP2A/ARRB2 complex. Abbreviations: AC, adenylate cyclase; AKT, protein kinase v-akt murine thymoma viral oncogene homolog; AP2, adaptor protein; ARRB2, β-arrestin 2; CME, clathrin-mediated endocytosis; DA, dopamine; DRD2, Dopamine D2 receptor; GSK-3, glycogen synthase kinase-3; GRK6, G protein-coupled receptor kinase-6.

The chronic excess blockade of DRD2 by antipsychotics induces a vicarious increase in DRD2 densities or in the proportion of D2^High^, probably due to alteration of the GRK6/ARRB2 system in patients with DSP. When the up-regulated DRD2 or D2^High^ receives endogenous DA, amplification of G protein-mediated early signaling in the downstream of DRD2 occurs at several stages of the cascade, which probably augment the signaling in a synergistic manner. In addition, DRD2 densities could gradually increase, even after cessation of antipsychotics, in an animal model [37,38]. Therefore, the medication nonadherence observed frequently in patients could affect DRD2 itself and the subsequent signaling more significantly than expected; in patients with DSP, a reduction or discontinuation of antipsychotics might induce a larger increase in available DRD2 for DA binding [18], and furthermore promote DA signaling compared with those without DSP. By the same token, this DSP process could be also more affected by the elimination half-life of antipsychotics, since DRD2 under agents with a short half-life are more easily available to endogenous DA. Consequently, DSP episodes, especially rebound psychosis, could present with unstable symptoms of disease (Figure 3). Furthermore, given that the GRK6/ARRB2 system is involved not only in DRD2 internalization but also in the DRD2-mediated effects of antipsychotics, DRD2-mediated effects of antipsychotics could wane. Therefore, a higher dosage of antipsychotics would be needed to control psychosis in patients with DSP, which is compatible with the clinical phenomenon of developed tolerance to antipsychotic effect. On the other hand, GSK-3, which plays an intensive role in DA signaling, maybe experience compensatory up-regulation under antipsychotic medications like those of DSP patients. This molecule or pathway might be related to the occurrence of psychotic relapses that appear immediately after treatment cessation or minor stress, since GSK-3 up-regulation potentiates ARRB2/AKT/GSK-3 DA signaling. Taken together, these compensatory up-regulations of DA signals could be fully responsible for the pathogenesis of DSP, although the precise cause of DSP has not yet been fully elucidated (Figure 4).

## 4. Conclusions

Although DSP or the DA supersensitivity state has been examined with a separate focus on withdrawal psychosis, relapsed episode, or TD in symptomatology and pharmacology research areas, data concerning the ultimate effect of DSP on long-term prognosis in patients experiencing DSP episodes remains scarce. However, the accumulating literature on this topic strongly supports a contributing role of DSP to treatment-resistance or refractoriness. A large part of pharmacogenomic research examines the genetic polymorphism of DRD2 and its responsiveness to treatment, demonstrating a significant strong relationship between them. Concurrently, DRD2 polymorphism is related to the expressed level of the receptor, and hence, it is possible that the polymorphism underlies the development of DA supersensitivity state or DSP. On the other hand, regarding the signal transduction downstream to DRD2, numerous studies suggest that molecules relevant to doperminergic signals are somehow involved in disease etiology or the action mechanism of antipsychotic(s). However, the roles of these proteins in DRD2 expression under administration of antipsychotics—their roles in developing DSP—have been little explored, and the essential etiology of DSP remains unknown. Since it is clinically considered that development of DSP is profoundly related to treatment-resistance, the mechanism of the DA supersensitivity state affected by antipsychotics will be very important in future schizophrenia research.
